# Multisystemic Sarcoidosis Revealed by Hepatosplenomegaly: A Case Report

**DOI:** 10.7759/cureus.23967

**Published:** 2022-04-08

**Authors:** Fatima Zahra El Rhaoussi, Soukaina Banani, Sophia Bouamama, Nissrine Bennani, Wafaa Badre

**Affiliations:** 1 Gastroenterology and Hepatology, Ibn Rochd University Hospital Center, Hassan II University of Casablanca, Faculty of Medicine and Pharmacy, Casablanca, MAR; 2 Anatomical Pathology, Ibn Rochd University Hospital Center, Hassan II University of Casablanca, Faculty of Medicine and Pharmacy, Casablanca, MAR

**Keywords:** multisystemic sarcoidosis, lymphadenopathy, corticosteroids., non-caseating granulomas, hepatosplenomegaly

## Abstract

Sarcoidosis is a systemic granulomatous disease of unknown etiology, characterized by the presence of non-caseating granulomas. Gastrointestinal involvement in sarcoidosis is extremely rare. However, hepatic sarcoidosis occurs in 70% of cases. This is a case report of multisystemic sarcoidosis revealed by hepatosplenomegaly. The patient presented initially with asthenia, anorexia, and weight loss. An abdominal computed tomography scan revealed hepatosplenomegaly and lumbo-aortic adenopathy. During hospitalization, the patient presented an extended erythematous cutaneous lesion in the peri-auricular area. The diagnosis of sarcoidosis was confirmed by salivary, cutaneous, and bronchoscopic biopsy, which revealed the presence of epithelioid granuloma without necrosis. Consequently, the patient was treated with oral corticosteroids with good improvement.

## Introduction

Sarcoidosis is a granulomatous disease of unknown etiology, involving many organs with a predilection for lungs, skin, eyes, and the reticuloendothelial system [1,2]. Since the clinical manifestations are usually not pathognomonic, diagnosis is generally based upon histology and occurs in approximately 75% of cases, non-caseating granulomas [1,3]. Two-thirds of patients have a resolution of symptoms in two years, in most cases spontaneously [4]. Pulmonary involvement occurs in 90% of cases, and extrapulmonary involvement exceeds 30% [1]. Gastrointestinal (GI) and hepatic involvement are extremely rare [3]. We presented a case of multisystemic sarcoidosis revealed initially by hepatosplenomegaly and abdominal lymphadenopathy.

## Case presentation

A 44-year-old woman with no relevant clinical background was hospitalized, having a five-month history of asthenia, anorexia, and weight loss with intermittent vomiting associated with dry cough. The initial physical examination revealed a hemodynamically stable patient with a blood pressure of 130/70mmHg, a pulse rate of 95/min, and a respiratory rate of 20/min. There were no rashes or skin lesions, no jaundice, and no palpable adenopathy. Cardiovascular and pulmonary examination findings were normal. The abdomen was flexible, not distended, with no tenderness to deep palpation, without hepatosplenomegaly or palpable mass. The remaining examination findings were unremarkable.

The blood test revealed abnormal liver function indicating elevated alkaline phosphatase (ALP) with a maximum value of 1157 U/L and gamma-glutamyl transpeptidase (GGT) elevated at 225 IU/L, lactate dehydrogenase of 427 U/L, liver enzymes were slightly increased, serum aspartate aminotransferase (AST) of 60U/L and alanine transaminase (ALT) of 46 U/L. C-reactive protein was elevated with a maximum value of 94.4 mg/L. The serological markers of hepatitis B virus and C virus were negative. Serum protein electrophoresis showed a polyclonal hypergammaglobulinemia.

**Figure 1:**
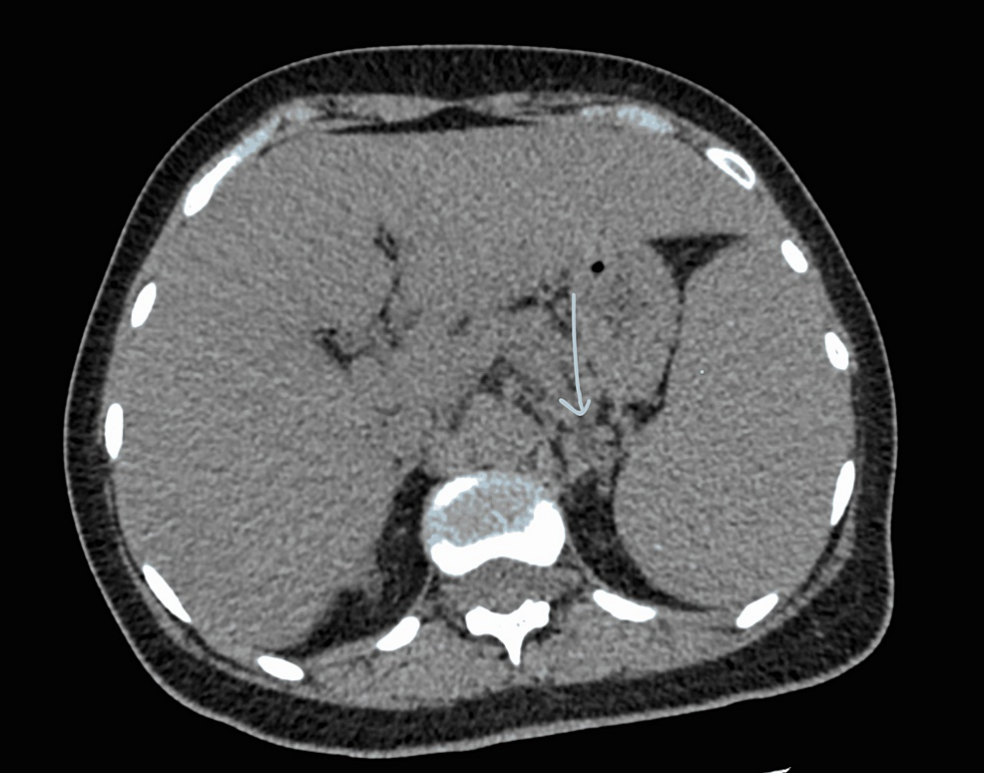
CT scan of the abdomen shows hepatosplenomegaly and abdominal lymphadenopathy

A body computed tomography scan showed bilateral micronodular pulmonary infiltrates showing multiple mediastinal adenopathies (the largest one measured 12 mm), slight homogeneous hepatosplenomegaly, and multiple lumbo-aortic adenopathies (Figure 1). The initial presentation of hepatosplenomegaly with multiple adenopathies raised the hypothesis of multifocal tuberculosis or neoplastic disease (lymphoma or gastric adenocarcinoma). Upper endoscopy revealed an aspect of extrinsic compression and gastritis. Histology of gastric biopsies showed nonspecific chronic inflammation with a high number of intraepithelial lymphocytes. An Xpert mycobacterium tuberculosis (MTB)/ rifampin (RIF; Cepheid Inc., Sunnyvale, USA)** **and three sputum samples were negative.

**Figure 2:**
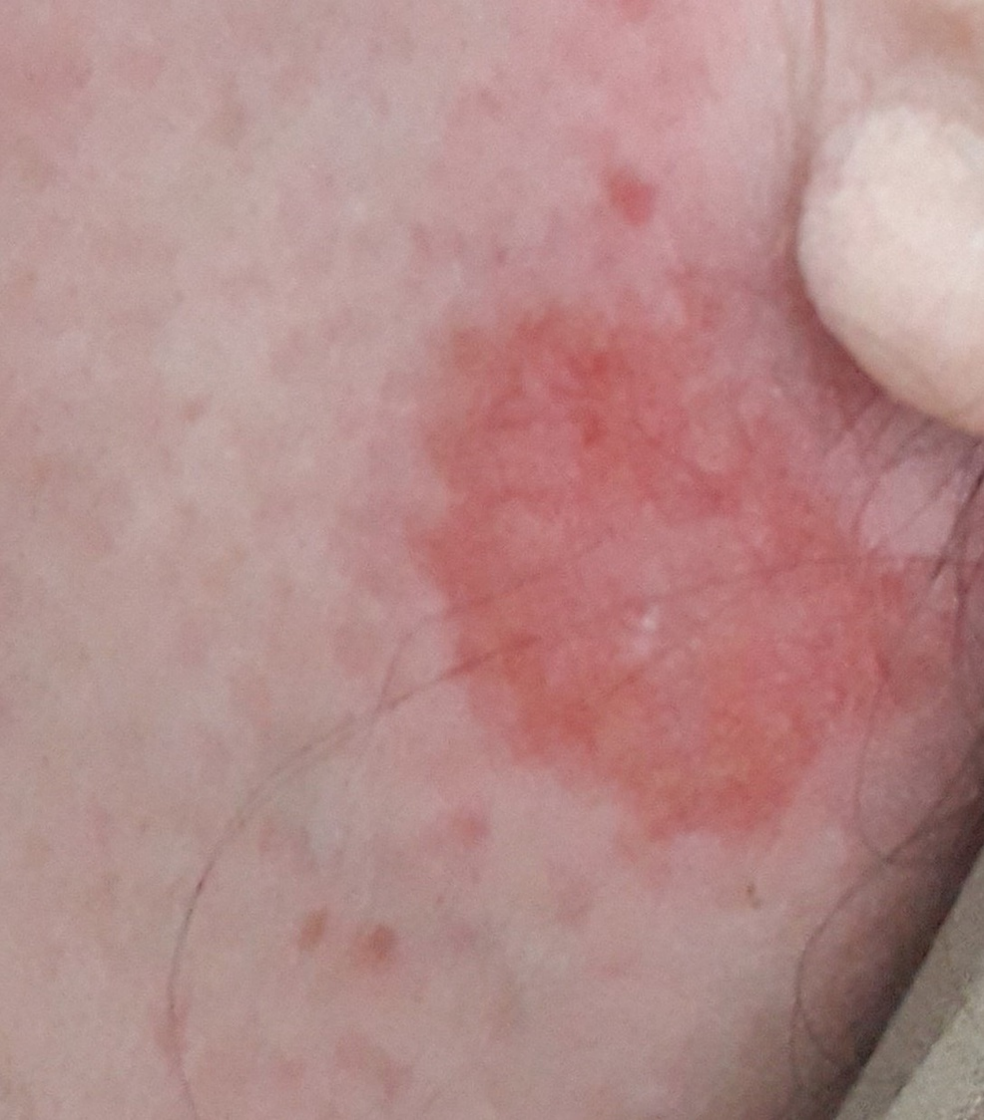
Extended erythematous cutaneous lesion with telangiectasia in the periauricular area

**Figure 3:**
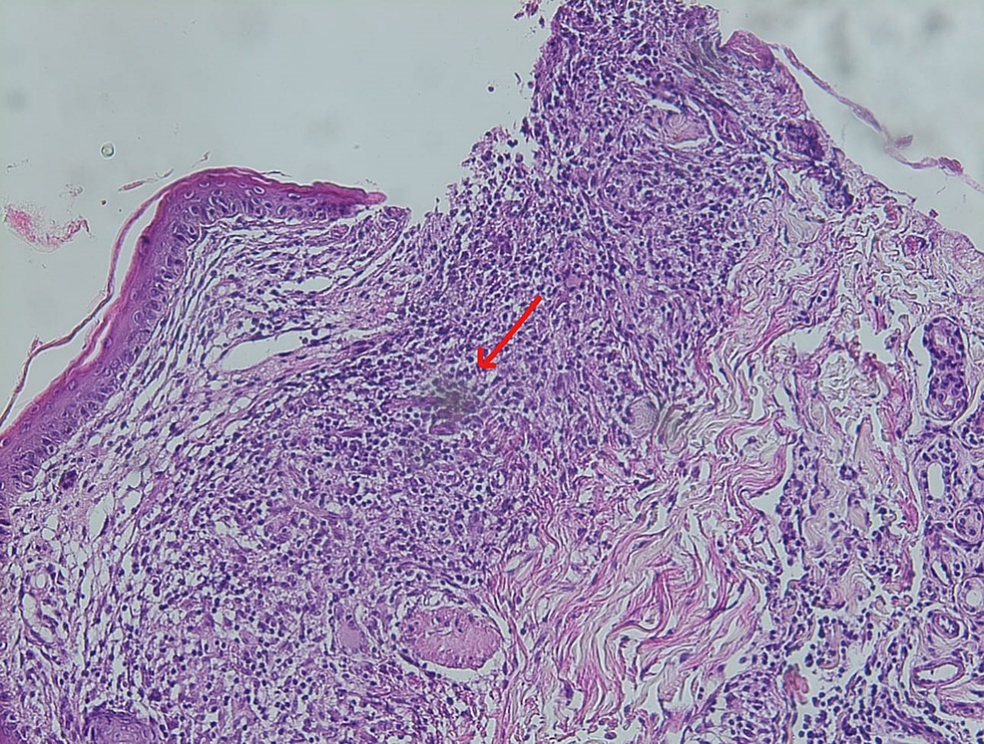
Cutaneous biopsy shows non-caseating granulomas without necrosis

During her hospital stay, the patient presented an extended erythematous cutaneous lesion with telangiectasia in the periauricular area (Figure 2). The cutaneous biopsy revealed the presence of epithelioid granulomas without necrosis (Figure 3). The angiotensin-converting enzyme (ACE) was elevated at >200U/L.

**Figure 4:**
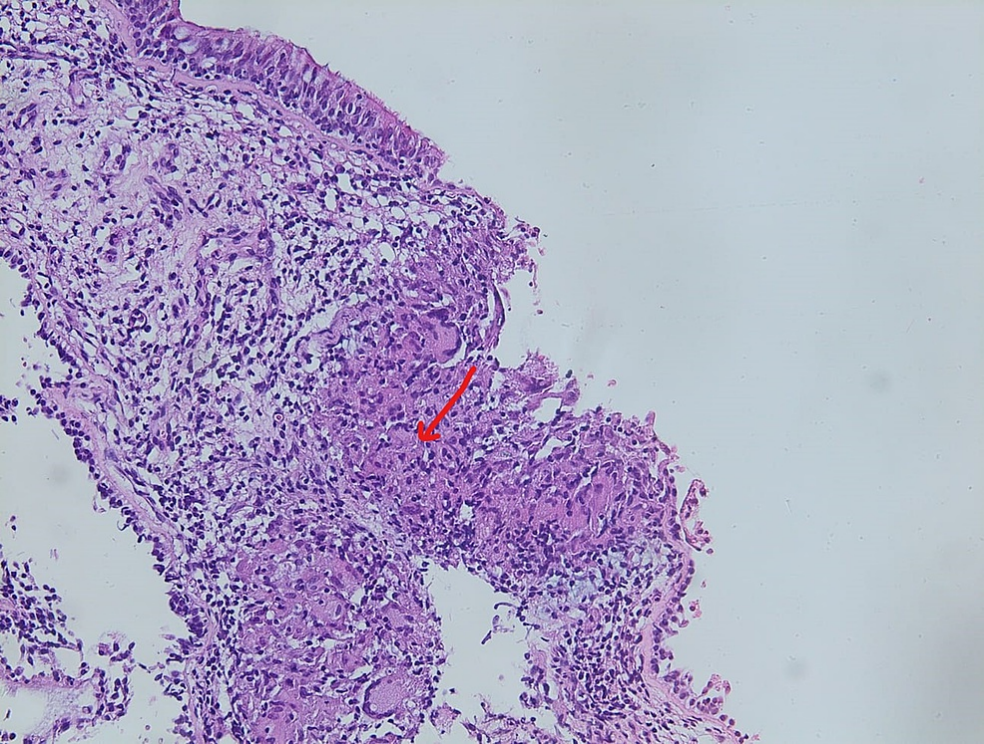
Bronchoscopic biopsy shows non-caseating granuloma without necrosis

Bronchoscopy showed an inflammatory condition with multiple granulations predominant in the lower lobar bronchus. Bronchoscopic biopsy showed an epithelioid granuloma without necrosis which is consistent with the diagnosis of sarcoidosis (Figure 4). We completed the investigations with accessible organs like the salivary gland, which also showed the presence of lymphocytic infiltration and an epithelioid granuloma with giant cells and without necrosis. As the diagnosis of systemic sarcoidosis was evident, the liver and lymphadenopathy biopsy was not performed. 

The diagnosis of multisystemic sarcoidosis was retained. The patient was treated with oral steroids: prednisolone 1 mg/Kg/day for eight weeks, then slightly digressed for two years. The evolution was marked by the amelioration of symptoms, regression of asthenia, weight gain, and dissipation of pulmonary symptoms. The liver function test improved remarkably after two months of treatment: the gamma-glutamyl transpeptidase (GGT) at 161 vs. 225 and alkaline phosphatase (ALP) at 131 vs. 1157, the lactate dehydrogenase (LDH) at 227 vs. 427 (Table 1).

**Table 1:** Liver blood tests before and after treatment GGT - gamma-glutamyl transpeptidase; ALP - alkaline phosphatase; LDH - lactate dehydrogenase

	Before treatment	After treatment
GGT	225 IU/L	161 IU/L
ALP	1157 U/L	131 U/L
LDH	427 U/L	227 U/L

## Discussion

Worldwide, sarcoidosis is seen more often in the northern European descent, while in the United States, African Americans are more likely to develop the disease and with more severe symptoms. It frequently occurs between the age of 20 and 40. Gastrointestinal involvement in sarcoidosis is extremely rare, especially with symptomatic manifestations; several autopsy studies found the gastric disease in 3.4% of cases [5]. The stomach, particularly the antrum, remains the most involved organ. The most common symptoms are abdominal pain, hematemesis, nausea, vomiting, and weight loss [6]. The last three symptoms were the only GI manifestations in our patient. The inspection of the gastric mucosa may appear normal, and multiple biopsies should be performed to make the appropriate diagnosis. Endoscopy often reveals gastritis, extrinsic compression (which were the findings in our patient), ulcers, nodular lesions, polyps, and thickening of the folds. Sarcoidosis of the esophagus, small bowel, and colon is the least common [3]. 

Hepatomegaly on abdominal computed tomography (CT) scan and abnormal liver function tests were the only hepatic manifestations present in our patient. Abnormal liver function tests are found in approximately 35% of cases, which is less common than histological changes and is independent of the degree of aggression and extent of the disease [7]. Hepatomegaly is clinically present in 21% of patients, while more than half of the patients have hepatomegaly on abdominal CT scans [8]. ALP is more reliable to predict liver involvement than GGT [9]. The histological abnormalities in liver sarcoidosis include non-caseating granulomas, chronic intrahepatic cholestasis, periportal fibrosis, and the development of micronodular biliary cirrhosis [10]. The clinical presentation of liver sarcoidosis extends from asymptomatic 'granulomas' to apparent hepatic disease with portal hypertension, intrahepatic, extrahepatic cholestasis, or both. Hepatic sarcoidosis can be responsible for Budd-Chiari syndrome [11], intrahepatic cholestasis with the development of biliary cholangitis, which mimics primary biliary cholangitis [12]. It is also similar to primitive cholangitis sclerosis due to the presence of portal and lobular non-caseating epithelioid cell granulomas surrounded by extensive fibrosis and bile duct proliferation with cholestasis [13].

The enlargement of the spleen is seen in about 5-14% of cases, most of which have intrathoracic involvement [14]. Splenomegaly in sarcoidosis is symptomatic in 15% of patients and is associated with hypersplenism in 20% of cases, especially with those having giant splenomegaly [15]. There is a correlation between ACE levels, extrathoracic sarcoidosis, and the size of the spleen [16]. Splenomegaly in sarcoidosis is associated with hepatomegaly and abnormal liver function in 86% of patients [17], including our case.

Glucocorticoids, which suppress the activated T helper type 1 ​​(TH1) cells lymphocyte processes responsible for the disease, remain the treatment of choice for sarcoidosis [17]. The major problem that clinicians face in treating sarcoidosis is choosing when to treat it because in about 50% of cases, the disease regress spontaneously. Generally, treatment with corticosteroids should be given in situations where some organ function is threatened, like lungs, eye, and central nervous system, and in diffuse cutaneous lesions [18]. The role of therapy in GI and hepatic sarcoidosis is unclear. However, corticosteroids may be beneficial in terms of improving liver function tests [19].

## Conclusions

In conclusion, we presented a case of multisystemic sarcoidosis with initial presentation of hepatosplenomegaly and GI manifestations, which raised the question of differential diagnosis of multifocal tuberculosis and neoplastic disease. The diagnosis was made by cutaneous, bronchoscopic, and salivary biopsy that showed an epithelioid granuloma without necrosis. The ACE was also elevated, which strengthened the diagnosis of sarcoidosis. The patient was treated with oral corticosteroids with good evolution. However, the therapy for hepatic and GI sarcoidosis remains a controversial topic.
